# Pleural effusion in pediatric patients submitted to liver transplantation: ultrasound and radiological assessment

**DOI:** 10.1186/2036-7902-6-S2-A10

**Published:** 2014-08-27

**Authors:** L Appierto, L Monti, Y Valzani, G Soglia, E Rossetti, R Bianchi, SG Picardo

**Affiliations:** 1 Ospedale Pediatrico Bambino Gesù, Roma, Italy

## Objective

To evaluate the ability of bedside lung ultrasound to diagnose and quantify pleural effusion in pediatric patients submitted to liver transplantation, in comparison with supine chest radiograph.

## Patients and methods

30 pediatric patients (mean age: 36 months, mean PIM2 11%) with end stage liver failure submitted to liver transplantation from living/dead donor (split). All the patients during the first three postoperative days were submitted to supine chest radiograph and bedside lung ultrasound, during mechanical ventilation or NIV.

Three measurements were made in first, second, and third postoperative day: chest x-ray was performed with a portable set; bedside lung ultrasound detected the pleural effusion at the PLAPS (posterolateral alveolar or pleural syndromes) point. The depth of the pleural effusion was evaluated according with the quad sign: the space outlined between the pleural line and the pulmonary line (indicating the visceral pleura).

Atelectasis, lung consolidation and pleural effusion are common in pediatric patients submitted to liver transplantation during the first postoperative days: chest radiograph showed to lack sensitivity in detecting pleural effusion and in differentiating atelectasis from pleural effusion, which is easy instead with lung ultrasound.

Ultrasound can detect the echogenicity of the effusion and help to assess the nature of it, measuring also its effective volume.

Statistically lung ultrasound has better sensitivity (80%) and specificity (90%), compared to chest radiograph, respectively 70% and 70%.

## Conclusion

Lung ultrasound is more sensitive and even more specific in comparison to chest radiograph to detect and measure pleural effusion in children submitted to liver transplantation.
